# Face Recognition Brain Functional Connectivity in Patients With Major Depression: A Brain Source Localization Study by ERP

**DOI:** 10.3389/fpsyt.2021.662502

**Published:** 2021-11-05

**Authors:** Lei Lei, Yu Zhang, Xiaotong Song, Penghong Liu, Yujiao Wen, Aixia Zhang, Chunxia Yang, Ning Sun, Zhifen Liu, Kerang Zhang

**Affiliations:** ^1^Department of Psychiatry, First Hospital of Shanxi Medical University, Taiyuan, China; ^2^Shanxi Medical University, Taiyuan, China

**Keywords:** major depressive disorder, face recognition, functional connectivity, source localization, ERP, EEG, face processing

## Abstract

**Objective:** Patients with major depressive disorder (MDD) presents with face recognition defects. These defects negatively affect their social interactions. However, the cause of these defects is not clear. This study sought to explore whether MDD patients develop facial perceptual processing disorders with characteristics of brain functional connectivity (FC).

**Methods:** Event-related potential (ERP) was used to explore differences between 20 MDD patients and 20 healthy participants with face and non-face recognition tasks based on 64 EEG parameters. After pre-processing of EEG data and source reconstruction using the minimum-norm estimate (MNE), data were converted to AAL90 template to obtain a time series of 90 brain regions. EEG power spectra were determined using Fieldtrip incorporating a Fast Fourier transform. FC was determined for all pairs of brain signals for theta band using debiased estimate of weighted phase-lag index (wPLI) in Fieldtrip. To explore group differences in wPLI, independent *t*-tests were performed with *p* < 0.05 to indicate statistical significance. False discovery rate (FDR) correction was used to adjust *p*-values.

**Results:** The findings showed that amplitude induction by face pictures was higher compared with that of non-face pictures both in MDD and healthy control (HC) groups. Face recognition amplitude in MDD group was lower compared with that in the HC group. Two time periods with significant differences were then selected for further analysis. Analysis showed that FC was stronger in the MDD group compared with that in the HC group in most brain regions in both periods. However, only one FC between two brain regions in HC group was stronger compared with that in the MDD group.

**Conclusion:** Dysfunction in brain FC among MDD patients is a relatively complex phenomenon, exhibiting stronger and multiple connectivity with several brain regions of emotions. The findings of the current study indicate that the brain FC of MDD patients is more complex and less efficient in the initial stage of face recognition.

## Introduction

Major depressive disorder (MDD) is a complex burdensome disorder and a major cause of functional disability across the globe. Its increased prevalence is attributed to the rapid development of society which increases pressure on daily life. Based on the World Health Organization (WHO), >264 million people of all ages suffer from depression (1), a major contributor to the overall global disease burden. Despite the effective treatments for mental disorders, 76–85% of people in low- and middle-income countries receive no treatment (2). In US adults, MDD is highly prevalent, where national 12-month and lifetime MDD prevalence is reportedly 10.4 and 20.6%, respectively (3).

Mechanisms of depression are quite complex and depressive symptoms might be affected by individual differences. As such, effective methods for early MDD diagnosis or clinical assessment are areas of intense research focus.

The common brain imaging techniques include electroencephalography (EEG), event-related potential (ERP), magnetoencephalogram (MEG), near-infrared spectroscopy (NIRS), functional magnetic resonance imaging (fMRI), and positron emission tomography (PET). EEG is a well-established technique of non-invasively mapping human brain function. As a bioelectrical signal that objectively reflects brain activity, EEG is popular in depression research because of its non-invasiveness, ease-of-use, and high time resolution. Numerous studies reported that brain activities of depression patients differ significantly from those of non-depressed people. As such, EEG can be used to evaluate brain activity in such patients (4–9).

Event-related potential (ERP) is potential changes to brain regions upon stimulation or withdrawal, or in response to psychological factors. When brain activity is induced by external stimulation, ERP reveals brain activity during the task. ERP is closely related to cognitive processing activities of the brain and is widely used in analyzing brain cognitive function.

The face plays a central role in many aspects of society (10). Face recognition is a highly developed skill in human beings due to its unique importance in social evolution. By glancing at a face, we can effortlessly gather information including age, sex, emotional state, and degree of concentration. Facial recognition and processing depends on a distributed network of brain regions; however, little is known concerning the interaction of these regions. Bruce and Young (11) subdivided face recognition and processing into two independent processes, i.e., facial recognition and facial emotion processing.

Most MDD studies have focused on emotional perception, including facial stimuli in emotions like happiness, surprise, anger, fear, sadness, and face neutrality (12–14). Nonetheless, relatively limited studies have systematically evaluated non-emotional aspects of face processing. Here, we used ERP analysis to explore the basic processes of face and non-face recognition stimuli.

Notably, brain function execution involves different brain regions. The brain network is the brain in task performance and is needed for several different functional areas of interaction and coordination to form a network executing corresponding functions. Based on different functions, brain connectivity is divided into structural connectivity (SC), functional connectivity (FC), and effective connectivity (EC) (15). FC is an undirected network describing the statistical connection between the nodes of the brain network. Functional and effective brain network research primarily uses imaging techniques to assess brain function, including EEG, MEG, and fMRI. Various studies reported a correlation between structural and FC alterations in MDD, yet what drives these changes remains unclear (16). In this study, we assessed any abnormal functional connection of face recognition in MDD.

Impairments in processing information from faces provide insights into the nature of social communication difficulties (17). Most ERP studies of face recognition and processing have focused on latency and amplitude changes of N170 and N200 but not changes in ERP components in other brain regions and periods. However, mechanisms of interaction in the brain regions to produce facial-processing specific capabilities are unclear. Numerous studies (18) identified a correlation between SC and FC changes in MDD yet what drives these changes remains puzzling. No comprehensive studies have investigated the enhanced or weakened N170 functional connectivity in the brain.

We sought to establish if relative to the normal population, face processing by depression patients differs in ERP brain network connection and a correlation functional connectivity state. For the first time, neural features of brain functional connectivity associated with face recognition was investigated in MDD patients vs. healthy participants by ERP. To explore the cognitive process of depressive patients, 64 lead EEGs were used to record differences between depressed patients and healthy participants using ERP. Besides, we assessed the potential functional connectivity abnormalities in MDD during face and non-face recognition tasks.

## Materials and Methods

### Participants

A total of 20 first-episode, drug-naive adult MDD patients (mean age 23.4 ± 9.433, 11 female) attending the Department of Psychiatry of the First Hospital of Shanxi Medical University between Sep 2019 and Jan 2020 were included in the current study. In addition, 20 matched healthy controls (HC) (mean age 25.15 ± 8.106, 10 female) were included in the study. Study participant features are summarized in Table 1. All participants were right-handed. Ethical approval for the study was obtained from the hospital ethics committee. All participants provided written informed consent. Two psychiatrists separately and independently evaluated medical and psychiatric history of participants through semi-structured interviews. Participants were interviewed using the Chinese version of the Modified Structured Clinical Interview for *DSM-5* patient version (19). MDD diagnosis was confirmed using the Diagnostic and Statistical Manual of Mental Disorders, Fifth Edition (DSM-5) (20). Additionally, depression symptoms were assessed using the 24-item Hamilton Depression Scale (HAMD-24) (21). All subjects met the following criteria: (1) age 18–55 years; (2) right-handedness; (3) no history of neurological illnesses or other severe diseases; (4) no physical trauma; and (5) no pregnancy or contraindications.

**Table 1:** Demographic characteristics of MDD patients and healthy participants.

	**MDD subjects** **(*****n*** **= 20)**		**Healthy controls** **(*****n*** **= 20)**		**x^**2**^/T**	***p*-value**
	**Mean**	**SD**	**Mean**	**SD**		
Sex, female	20 (11)		20 (10)		0.100	0.752^a^
Age, years	23.400	9.433	25.150	8.106	−0.629	0.533^b^
Years of education	11.800	2.764	13.250	2.653	−1.692	0.099^c^

a *p-Value for chi-square test*.

b *p-Values for double sample t-test*.

c *p-Values for double sample t-test*.

### Procedure

During the test and ERP signal acquisition, subjects were seated on a chair in front of a computer screen and remained relaxed, awake, and focused all the time in a bright, quiet room. As an experiment task, subjects were asked to look at the screen, watch pictures on the screen, and make a quick judgment. If a face picture was presented, they pressed key “C.” For non-face pictures, the pressed key “M.”

### Experimental Design

**Figure 1:**
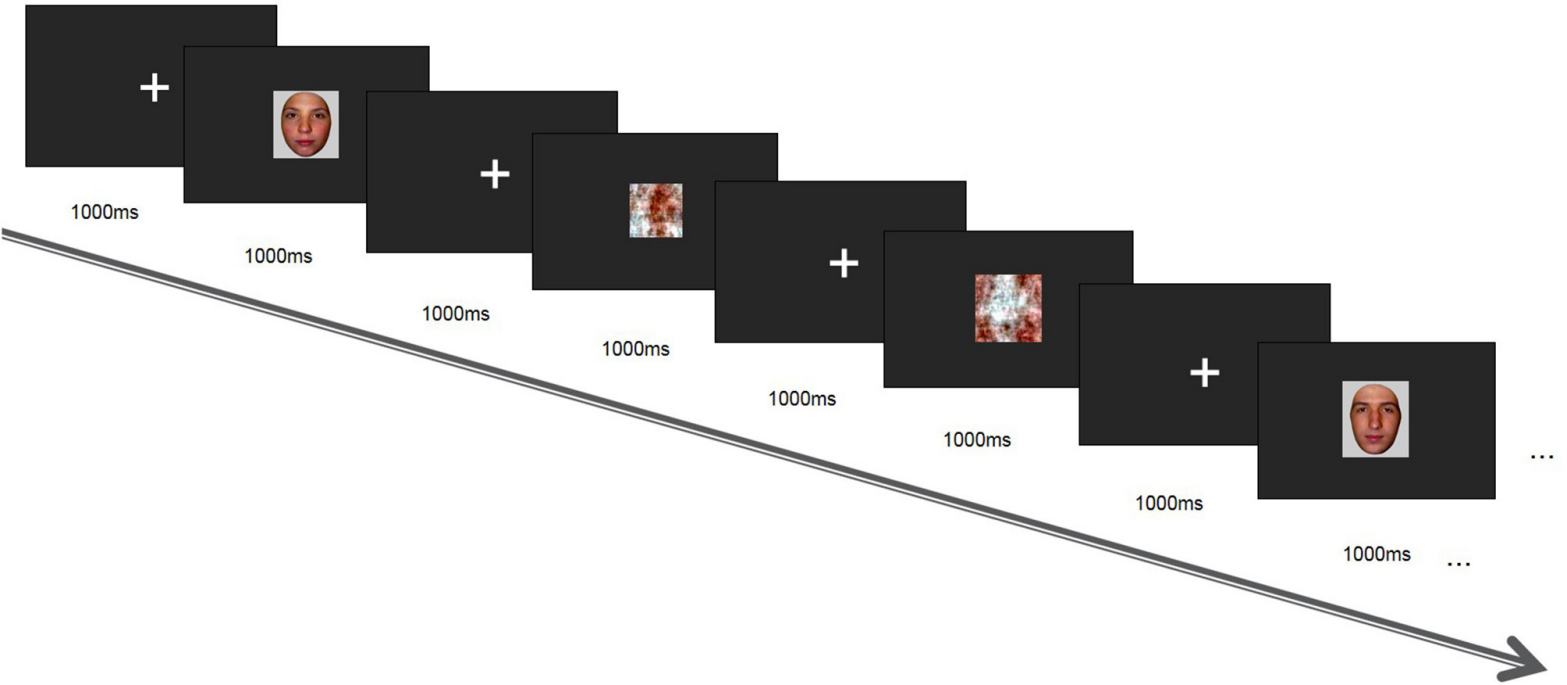
Presentation phase of face recognition task.

This task was based on face recognition. Stimulus material was presented using E-prime software. Visual stimulation comprised 10 cm × 7 cm face and landscape pictures selected from the international emotional picture system. The stimulus sequence in the task paradigm comprised two stimuli types, including neutral face images (80) without any emotions as target stimuli and non-face images (80) as non-target stimuli. The total number of stimuli was 160, and the probability of occurrence was 50. All stimuli were randomly presented. Visual stimuli appeared immediately after a prompt (cross) at the center of the screen (Figure 1). All stimuli appeared randomly at the center of the display screen, with each picture shown for 300 ms, at a picture interval of 1,000 ms. Total task time was ~8 min. Subjects were tasked with looking at the screen and pressing “C” when a face picture appeared, or “M” in case of a non-face picture.

### ERP Recording

**Figure 2:**
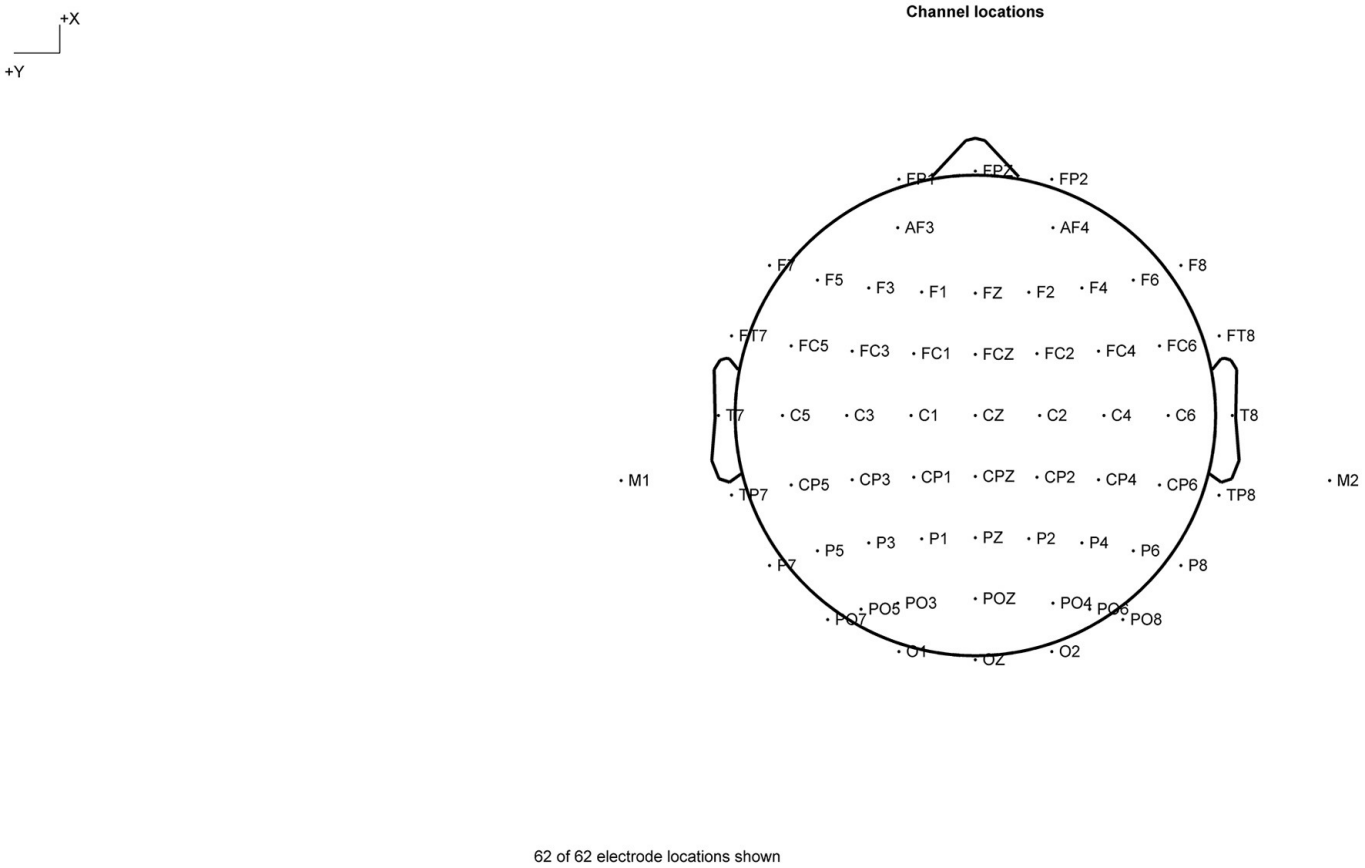
Channel locations.

ERPs were recorded from a 64-channel/electrodes system using the Neuroscan acquisition system (Compumedics, Abbotsford, VIC, Australia). The 64-Ag/AgCL electrodes cap based on the international 10–20 system is shown in Figure 2. The reference electrode was placed on the binaural mastoid (M1, M2) and the parietal lobe was positively positioned on the ground electrode (GND). Resistance of reference electrodes to scalp lead was <5 kQ. Data were sampled at 1,000 Hz; referenced to the M1, M2 electrode; and collected for offline processing.

### Data Pre-processing

EEG data analysis was conducted using custom-developed Matlab (22) scripts (v.R2013b; MathWorks, Natick, MA, USA) and EEGLAB (23). Band-pass filters were set at 0.1–70 Hz with an additional Notch filter at 50 Hz to remove line noise. EEG data were first down-sampled to 250 Hz then divided into segments, taken 0.1 s before and 1 s after face or no face stimulus onset. Thus, the segmented time window was −1,000 to 1,000 ms. Epochs were baseline-corrected. EEG deflections resulting from eye movements, blinks, and muscle artifacts were corrected using an independent component analysis (ICA) procedure. The remaining artifacts exceeding 100 mV in amplitude were rejected.

### Data Processing

#### EEG Source Reconstruction

Spatiotemporal distribution of cortical source activations was reconstructed using the Fieldtrip toolbox (24). EEGLAB-preprocessed data were converted into Fieldtrip format and processed in Fieldtrip by loading the default head mold standard in Fieldtrip BEM using one data to build forward solutions (a forward model). Noise was regressed in the time dimension for each trial in ERP. The selected period was −1,000 to 1,000 ms. The inverse solution was computed using the minimum-norm estimate (MNE) (25). Source reconstruction was calculated based on brain average. After source reconstruction, data from each trial were interpolated, smoothed, and recut to the AAL90 template to obtain a time series of 90 brain regions, where source activations of single trials were modeled.

#### Functional Connectivity

EEG power spectra were calculated using Fieldtrip incorporating a Fast Fourier transform (4–70 Hz, at a frequency resolution of 0.5 Hz). Connectivity was calculated across all pairs of brain signals for each frequency band using the debiased estimate of weighted phase-lag index (wPLI) in Fieldtrip (24). This method was selected since it prevents volume conduction effects from affecting the outcomes, whereas the wPLI provides a conservative and reliable estimate of phase synchronization, and robust to any noise within the dataset (26–28). Then, power values were averaged across theta frequency band (4–7 Hz) and averaged across all trials. Results were then averaged across each frequency band, resulting in a weighted matrix of undirected connectivity strengths for each separate frequency band for every subject.

### Statistical Analysis

Statistical analyses were performed using MATLAB, EEGLAB, and Fieldtrip. To evaluate group differences in wPLI, we ran independent *t*-tests, with *p* < 0.05 indicating statistical significance. False discovery rate (FDR) correction was used to adjust *p*-values.

## Results

Through time-domain analysis, the ERP amplitudes of depression patients and healthy controls were compared from −0.2 to 1.0 s after stimulation.

**Figure 3:**
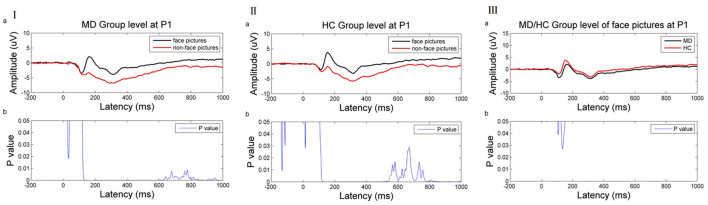
**(I)** ERP results of the MDD group at the P1 electrode. **(II)** ERP result of HC group at the P1 electrode. **(III)** ERP result of MD/HC group of face picture stimulus at the P1 electrode. (a) Average ERP results for the MD/HC group between −200 and 1,000 ms. The horizontal axis represents the time axis and the vertical axis represents the amplitude. (b) Difference between two kinds of stimuli during this period. The horizontal axis represents time, and the vertical axis represents *p*-value range. The blue line indicates the time range when the p value of this difference is <0.05.

Time-domain analysis revealed a significant difference between face recognition and non-face recognition in depression patients between 120 and 1,000 ms (*p* ≤ 0.05). Amplitude induction by face pictures was higher than that of non-face pictures [e.g., at the P1 electrode (Figure 3I)].

Similarly, a significant difference was noted between face recognition and non-face recognition in the normal control group between 106 and 1,000 ms (*p* < 0.05). As in MDD patients, the induced amplitude by face pictures was higher than that of non-face pictures [as shown at the P1 electrode (Figure 3II)].

Comparison of face recognition amplitude in patients with depression vs. health controls found that face recognition amplitude in MDD patients was lower than in healthy controls, but the difference was significant only in 105–112 and 126–151 ms [as shown at P1 electrode (Figure 3III)].

Based on the above results, this work selected these two time periods and compared functional connectivity differences in face recognition in the depression group vs. the normal group after EEG source localization. Theta band (4–7.5 Hz) was related to emotional processing. Thus, wPLI values were compared in the theta band and 105–112 and 126–151 ms periods.

**Figure 4:**
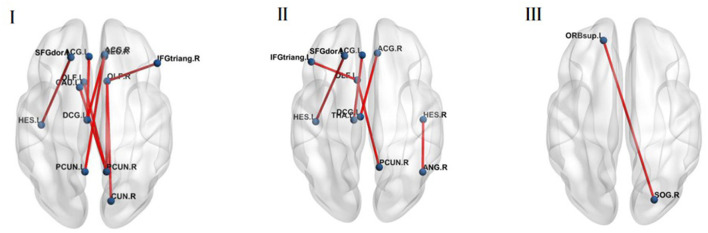
**(I)** The MDD group was stronger compared with the HC group in the brain regions of theta band functional connectivity at 100–110 ms (*p* < 0.05, FDR corrected). **(II)** The MDD group was stronger compared with the HC group in the brain regions of theta band functional connectivity at 120–150 ms (*p* < 0.05, FDR corrected). **(III)** The MDD group was weaker compared with the HC group in the brain regions of theta band functional connectivity at 120–150 ms (*p* < 0.05, FDR corrected).

At 100–110 ms, theta band functional connectivity in the MDD group was stronger than in the HC group in the following brain regions: right olfactory cortex with right cuneiform lobe (*t* = −3.488, *p* < 0.001, *FDR* corrected) and right inferior frontal gyrus of trigone (*t* = −3.763, *p* < 0.001, *FDR* corrected), right precuneus with bilateral olfactory cortex (left: *t* = −4.201, *p* < 0.001, *FDR* corrected; right: *t* = −3.763, *p* < 0.001, *FDR* corrected), left caudate nucleus (*t* = −3.774, *p* < 0.001, *FDR* corrected), left precuneus with right rectus gyri (*t* = −3.552, *p* < 0.001, *FDR* corrected), the left medial and paracingulate gyri with the bilateral anterior and paracingulate gyri (left: *t* = −4.037, *p* < 0.001, *FDR* corrected; right: *t* = −4.781, *p* < 0.001, *FDR* corrected), and left transverse temporal gyrus and left dorsolateral superior frontal gyrus (*t* = −3.667, *p* < 0.001, *FDR* corrected; Figure 4I).

At 120–150 ms, the MDD group was stronger than the HC group in the following brain regions: left olfactory cortex with the right precuneus (*t* = −3.502, *p* < 0.001, *FDR* corrected) and the left inferior frontal gyrus of the trigone (*t* = −3.662, *p* < 0.001, *FDR* corrected), left anterior cingulate gyrus with paracingulate gyrus and left thalamus (*t* = −3.499, *p* < 0.001, *FDR* corrected), right anterior cingulate gyrus and right lateral cingulate gyrus with left medial cingulate gyrus and right lateral cingulate gyrus (*t* = −4.321, *p* < 0.001, *FDR* corrected), left transverse temporal gyrus and left dorsolateral superior frontal gyrus (*t* = −3.525, *p* < 0.001, *FDR* corrected), right transverse temporal gyrus and right angular gyrus (*t* = −3.604, *p* < 0.001, *FDR* corrected; Figure 4II).

However, functional connectivity between the right superior occipital gyrus and the left superior orbital frontal gyrus in normal people was higher than in MDD patients (*t* = 3.602, *p* < 0.001, FDR corrected) (Figure 4III).

## Discussion

The human face is an important form of social stimulation and the basis of social communication. People transmit basic social information including identity, expression, and intention through the face and its recognition, an important form of interpersonal social communication. We found that the brain functional connectivity of MDD group was more complex than HC group during the early stage of face recognition.

### Difference in ERP Amplitudes

Accumulating evidence shows that MDD patients have face recognition and processing disorders (29, 30). Previous research identified N170 as a face-specific ERP component, a negative wave detected in the occipitotemporal region between 130 and 190 ms after facial stimulation. It reflects the structural coding of face processing (15, 31). The N170 amplitude of human face stimulations is larger than that of non-face stimulations and is also called the N170 effect (32). It reflects the early detection of facial information which distinguishes faces from non-faces. Electrophysiological specificity of face recognition may lie in the special facial processing owing to the N170 of the face different from the N170 of the object. The findings from the first part of ERP analysis are consistent with previous findings which report that object and face processing differ as early as at 120 ms. The findings showed that the N170 amplitude induced by face recognition in the MD and HC groups was higher than that of non-face images.

Comparison of the N170 amplitude of the MD group with the HC group showed that the N170 amplitude induced by facial information stimulation in the depression group was significantly lower relative to the control group. This indicated an abnormal early perceptual processing of facial information in depression patients, in line with previous findings. Moreover, Chen et al. (33) reported that relative to the control group, the N170 amplitude of first-episode depression patients identifying positive, neutral, and negative emotional pictures decreased. Face processing is a complex cognitive process comprising visual processing of face information, face recognition, identity recognition, expression analysis, and other cognitive processing stages (11). Therefore, visual processing of face information is the early perceptual processing of physical features of face stimulation by the human brain, which excludes processing of other features such as identity recognition or expression analysis. Therefore, impairment of facial information processing ability in depression patients might occur in the early stage of face recognition, rather than in the stage of emotional processing recognition.

### Brain Functional Connectivity During Face Recognition

An abnormal N170 index might be a characteristic marker for depression patients, and its electrophysiological mechanism might be the neural basis of clinical symptoms of depression (34). The theta band participating in emotional processing is seemingly a good feature in diagnostic tools (35–43). However, information on its mechanisms is limited. Here, we used to explore changes in brain functional connectivity during face processing.

In the initial 100–110 ms of stimulation, the MDD group was stronger than the HC group in the following aspects; right olfactory cortex and right cuneiform lobe, right inferior frontal gyrus of triangle, bilateral olfactory cortex, and right precuneus lobe. The findings for the current study indicate that depressed patients potentially have excessive enhancement in this circuit. The olfactory cortex is located next to the hippocampal formation. The olfactory cortex includes the entorhinal cortex and perirhinal cortex. The most direct sensory input of the olfactory cortex comes from vision. Precuneus is a part of the posterior parietal cortex, located in the inner hemisphere of the brain. Its cognitive functions include episodic memory, visual space, self-related information processing, cognition, consciousness, and other processes. Face selectivity also extended into the precuneus (44). In the entorhinal cortex, as the transfer station of information input and output in the precuneus, the Cuneiform leaf facilitates information exchange between the hippocampus and the cerebral cortex. These outcomes imply that depression patients might develop excessive enhancement in this loop. Similarly, MDD patients show repeated abnormal activation patterns in parts of the cingulate gyrus and orbitofrontal cortex (45). The cortico-striatal-pallidal-thalamic and limbic circuits regulate the pathophysiology of depression (46).

Furthermore, the FC between the left precuneus, right rectus gyri, right precuneus, and left caudate nucleus in the MDD group was stronger compared with those in the HC group in the initial 100–110 ms of stimulation. The FC between the left medial and paracingulate gyrus and bilateral anterior cingulate gyrus and paracingulate gyrus was higher in the MD group compared with that in the HC group. In the period between 120 and 150 ms, FC in the MDD group was stronger compared that in the HC group. This included the left olfactory cortex, right precuneus lobe and left inferior frontal gyrus, left anterior cingulate gyrus, paracingulate gyrus and left thalamus, right anterior cingulate gyrus and right lateral cingulate gyrus, left medial cingulate gyrus and right lateral cingulate gyrus, left transverse temporal gyrus and left dorsolateral superior frontal gyrus, and right transverse temporal gyrus and right angular gyrus.

Regarding the theory of face recognition, Duchaine and Yovel (47) suggested a revised neural framework for face processing (48), dividing the network of face-selective areas into two streams (49). One is a ventral stream extracting information from faces while the other is a dorsal stream specialized in processing dynamic face information (50, 51). The ventral pathway includes the occipital face area (OFA), fusiform face Area (FFA), and anterior temporal lobe-face area (ATL-FA). The dorsal pathway comprises the posterior superior temporal sulcus-face area (pSTS-FA), anterior superior temporal sulcus, and inferior frontal gyrus-face area (IFG-FA). Davies-Thompson and Andrews (44) reported consistent face selectivity in core face regions of the occipital and temporal lobes, i.e., FFA, OFA, and superior temporal sulcus. Face selectivity extended into the intraparietal sulcus, precuneus, superior colliculus, amygdala, and inferior frontal gyrus. We also found that at 100–110 and 120–150 ms, functional connections between the fusiform gyrus and other brain regions were significantly different in MDD patients vs. HC, but not corrected by FDR. Only functional connections between the left transverse temporal gyrus and the left dorsolateral prefrontal cortex (DLPFC) in the MDD group were stronger than that in the HC group corrected by FDR. Notably, the temporal lobe is closely associated with human emotion and mental activity. In addition, DLPFC is involved in complex processing of emotions and plays a key role in pathology and physiology of depression (52). A previous study reported that information failed to propagate up the cortical-striatal-pallidalthalamic circuit to the DLPFC for contextual processing and reappraisal among patients with depression, and activated more of the limbic system involved in emotion (53). This finding implies that MDD patients overreacted to neutral face information which is involved in emotional brain regions. This may trigger decreased brain functional efficacy and energy depletion. Moreover, the effect of DLPFC regulates the deep structure of neural networks (54). These findings from previous studies inspired further studies to explore the deep brain network and function.

Therefore, we speculate that in the early stage of face recognition, MDD patients have more connections and greater intensity of brain regions related to emotion and face processing, even under the stimulation of neutral face. Nonetheless, the functional connectivity between the right superior occipital gyrus and the left superior orbital frontal gyrus in healthy people was stronger than that in MDD patients. The right superior occipital gyrus and the left superior orbital frontal gyrus, a network of face-selective areas, belonged to the ventral stream (49), i.e., the face recognition of healthy people was more direct and efficient, without adding more emotion-processing brain regions.

EEG measurements provide data with high temporal but low spatial resolution. In contrast, fMRI has high spatial resolution but low temporal resolution. EEG and fMRI provide complementary information about neural activity, but the two measurements are not commonly applied to evaluate face processing in MDD patients. Limited studies have examined the relationship between fMRI and ERP measures in normal individuals. Using EEG-fMRI to examine face and chair stimuli, Sadeh et al. (55) reported a strong association of ERP 170 ms with the mid-temporal areas, the FFA, and pSTS-FA, but not with the OFA. Previous studies examined the functional connectivity of fMRI/MEG for different emotional face processing and recognition in patients with depression. However, few studies have investigated the functional connectivity between face and non-face tasks in patients with depression. One previous study used fMRI to assess FC alterations based on neutral face and scene as the task stimuli to perform the task of visual delayed recognition. Their results demonstrated disruption of working memory updating in MDD by altered activity patterns in connectivity of the prefrontal cortex correlating well with core clinical characteristics (56). A review that included 25 studies using fMRI reported abnormal activation patterns in parts of the cingulate gyrus and the orbitofrontal cortex in MDD patients, as shown by functional connectivity analysis (57). Notably, the findings of the current study were not consistent with these findings. Further, the application of simultaneous ERP-fMRI to analyze face-selective mechanisms revealed that these complementary neural markers were highly correlated (58). Data presented above indicate that faces elicit highly selective fMRI and electrophysiological responses. Measurements obtained by fMRI and EEG regarding face processing were strongly correlated.

In conclusion, our data show that depression causes disorders of facial information processing. The finding showed that normal people process neutral face information through direct communication between the visual center of the superior occipital gyrus and the superior frontal gyrus of orbit. Nevertheless, dysfunction in brain functional connectivity in patients with depression is a relatively complex phenomenon, exhibiting stronger and multiple connectivity with many brain regions of emotions and face recognition. This indicates that the initial stage of face recognition is disrupted in patients with depression. The findings of the current study indicate that the brain network connection of MDD patients is significantly strong, and is attributed to reorganization of brain resources compared with that of health people. In addition, MDD patients activate more brain regions and connectivity unrelated to task information thus involving more emotion-related brain regions in neutral face recognition.

## Conclusion

The current study explored whether the functional connectivity of face recognition is enhanced or weakened in the brain. Moreover, facial processing in brain functional connectivity was compared between depression patients and healthy controls. In summary, the brain functional connectivity of patients with depression is more complex and less efficient in the initial stage of face recognition.

## Limitations

Several functional connections among key brain regions were observed in patients with depression and healthy controls; however, connections were not corrected by FDR. Furthermore, despite several converging findings, some findings were not consistent with findings from previous research, which is potentially caused by heterogeneities in paradigms and patient samples. Therefore, the functional roles and interactions among face-selective areas, mainly in MDD patients should be further investigated using larger sample sizes.

In the current study, only EEG data was used, and there was no collection and direct combination with fMRI data. Therefore, further studies should combine the 2 imaging methods (EEG and fMRI) to verify the findings from the current study.

The study did not carry out evaluation of clinical symptoms or cognitive function evaluation; thus, there was no related analysis. Further studies should include intact depression symptoms and cognitive function tests to explore the relationship between clinical symptoms or other cognitive functions of patients with depression.

## Data Availability Statement

The original contributions presented in the study are included in the article/supplementary material, further inquiries can be directed to the corresponding author/s.

## Ethics Statement

The studies involving human participants were reviewed and approved by Ethics Committee for Medicine of the First Hospital of Shanxi Medical University. Written informed consent to participate in this study was provided by the participants' legal guardian/next of kin. Written informed consent was obtained from the individual(s), and minor(s)' legal guardian/next of kin, for the publication of any potentially identifiable images or data included in this article.

## Author Contributions

LL and KZ designed the experiment. PL, YW, AZ, CY, NS, and ZL carried it out. LL and YZ analyzed the data. LL wrote the manuscript. YZ and XS edited the manuscript. All the authors discussed the results and reviewed the manuscript. All authors listed have made a substantial, direct and intellectual contribution to the work, and approved it for publication.

## Funding

This work was supported by the National Natural Science Youth Fund Project (81701345), a research project supported by the Shanxi Scholarship Council of China (HGKY2019098), and Natural Science Foundation of Shanxi Province for youth (201601D021151).

## Conflict of Interest

The authors declare that the research was conducted in the absence of any commercial or financial relationships that could be construed as a potential conflict of interest.

## Publisher's Note

All claims expressed in this article are solely those of the authors and do not necessarily represent those of their affiliated organizations, or those of the publisher, the editors and the reviewers. Any product that may be evaluated in this article, or claim that may be made by its manufacturer, is not guaranteed or endorsed by the publisher.
